# Unravelling taboos and cultural beliefs associated with hidden hunger among pregnant and breast-feeding women in Buyende district Eastern Uganda

**DOI:** 10.1186/s13002-024-00682-z

**Published:** 2024-05-02

**Authors:** Patience Tugume, Abubakar Sadik Mustafa, Abdul Walusansa, Samuel Ojelel, Evelyne B. Nyachwo, Emmanuel Muhumuza, Maria Nampeera, Fredrick Kabbale, Jamilu.E. Ssenku

**Affiliations:** 1 Department of Plant Sciences, Microbiology and Biotechnology, College of Natural Sciences, Makerere University, P. O. Box 7062, Kampala, Uganda; 2 Department of Health Policy Planning and Management, School of Public Health, Makerere University, P. O. Box 7072, Kampala, Uganda; 3 Department of Medical Microbiology, Habib Medical School, Faculty of Health Sciences, Islamic University in Uganda, P. O. Box 7689, Kampala, Uganda; 4 Department of Health Nutrition, Buyende District Local Government, P. O. Box 87, Kamuli, Uganda; 5 Directorate of Research and Quality Assurance, Busoga University, P. O. Box 154, Iganga, Uganda; 6 Department of Production, Buyende District Local Government, P. O. Box 87, Kamuli, Uganda

**Keywords:** Micronutrient deficiency, Maternal health, Food taboos, Cultural beliefs, Breast-feeding women

## Abstract

**Background:**

Food taboos and cultural beliefs among pregnant and breast-feeding women influence their food consumption patterns and hence the health of women and unborn children. Cognizant of their neglect in programs aimed to ameliorate hidden hunger among pregnant and breast-feeding women in Buyende and other resource-poor communities in sub-Saharan Africa, we opted for a study to unravel them to inform program design.

**Methods:**

We documented food taboos and beliefs amongst pregnant and breast-feeding women from six sub-counties of Buyende district in Eastern Uganda. A mixed-methods approach was used, which was comprised of questionnaire interviews with 462 women, eight focus group discussions with 6–10 participants in each and a total of 15 key informant interviews.

**Results:**

The present study revealed that 129 (27.9%) of the respondents practice food taboos and adhere to cultural beliefs related to their dietary habits during pregnancy and breast-feeding that are fuelling the prevalence of hidden hunger. The most tabooed foods during pregnancy were sugarcane (17.8%), fishes which included lung fish, catfish and the Lake Victoria sardine (*Rastrineobola argentea*) (15.2%), oranges (6.6%), pineapples (5.9%), eggs (3.3%), chicken (3.3%) and cassava, mangoes and *Cleome gynandra* (each at 3%). Most foods were avoided for reasons associated with pregnancy and labour complications and undesirable effects on the baby. Most women learnt of the taboos and beliefs from the elders, their own mother, grandparents or mother-in-law, but there was also knowledge transmission in social groups within the community.

**Conclusions:**

The taboos and cultural beliefs in the study area render pregnant and breast-feeding women prone to micronutrient deficiency since they are denied consumption of a diversity of nutritious foods. There is a need to educate such women about consumption of nutrient-rich foods like fish, eggs, fruits and vegetables in order to improve their health, that of the unborn and children being breast fed. Additionally, culturally appropriate nutrition education may be a good strategy to eliminate inappropriate food taboos and beliefs with negative impact on the health of pregnant and breast-feeding women.

## Background

Hidden hunger is a widespread problem in developing countries, characterized by lack of essential vitamins and minerals nutrients in the diet, despite adequate calorie intake [1]. Globally, approximately two billion people especially in low-and middle-income countries experience hidden hunger [2, 3] that has for long been recognized as a public health problem, particularly among vulnerable groups including pregnant and breast-feeding women [4]. In pregnant and breast-feeding women, it has been observed to cause intrauterine growth restriction which adversely affect foetal development, energy production, birth weight, blood formation, brain development, maternal mortality and prenatal complications [5, 6]. Approximately 800 women die from pregnancy or childbirth-related complications around the world every day [7] with almost all the deaths occurring in areas of low-resource settings in developing countries [8] that are predominantly characteristic of rural communities in Uganda, Buyende inclusive. Uganda is ranked among the 10 leading countries with maternal death in the entire world [9]. Some studies have reported practicing of food taboos by pregnant and breast-feeding women among relatively poor communities in sub-Saharan Africa and harmful effects of tabooed foods to the newborn [10, 11]. High prices of some food like meat, fish and eggs which are protein-rich cannot be accessed by poor communities. For instance, an increase in income is more associated with regular meat consumption in Ethiopia [12]. Thus, taboos can exacerbate the problem of undiversified diet among pregnant and breast-feeding women from poor households. The uneducated often have limited sources of income and thus may be unable to afford diverse diets. However, educated women who may be employed are more likely to afford diversifying their diets during pregnancy and breast-feeding. Furthermore, education equips women with knowledge about positive effects of a balanced diet compared to the uneducated. Such knowledge is gained from formal education and health education during antenatal visits. Regardless of the importance of education, Ali et al. [13] and Patil et al. [14] reported the same misconceptions of dietary practices from the educated and uneducated pregnant and breast-feeding women. Age, marital status and occupation of women also influence their adherence to food taboos during pregnancy and breast-feeding. Younger women below 20 years are more likely to attend antenatal care and health education programmes compared to older women that tend to believe more in indigenous knowledge of traditional practices [15]. Old women are thus more likely to adhere to food taboos. Young women easily accept modern health services and are likely to abandon food taboos. In Ethiopia young educated mothers who attended antenatal clinics reported attaching value to a balanced diet during pregnancy [16]. Another study reported that hat elderly women adhered more to food taboos than younger women [17]. Being employed is a major determinant of a positive pregnancy outcome [18]. Some studies have reported a link between poverty and malnutrition during pregnancy [19]. The poor are unable to afford the minimum required food intake [20] given the persistent rise on food prices. However, Ramulondi et al. [18] revealed a difference in food avoided or consumed by the employed and unemployed. Thus, poverty may not contribute towards dietary intake during pregnancy in relation to food taboos. Marital status was statistically associated with the likelihood of observing food taboos [21]. The same study reported less likelihood of single and widowed women observing tabooed foods than married women. This was attributed to the patriarchal tendency in most developing countries where women are expected to abide and respect ideas and beliefs of their husbands.

Women’s health, nutrition and well-being across the continuum of preconception, pregnancy and postpartum are critical for ensuring desirable pregnancy outcomes and long-term benefits for both mothers and their offspring [22, 23]. Maternal malnutrition is not solely a consequence of inadequate nutrition intake but it is also influenced by social and psychological factors, nutritional knowledge and biological changes that impact dietary patterns during pregnancy [8, 23]. Therefore, attention to appropriate dietary behaviour and proper nutrient intake is fundamental for adequate nourishment to both the mother and the foetus. Lack of accurate information concerning food intake of pregnant women could hinder efforts to enhance their nutritional well-being.

Taboos that prohibit the consumption of specific foods for sociocultural or religious reasons have been reported as one of the contributors to hidden hunger worldwide [24]. Religious beliefs strongly influence food taboos by declaring some food items unfit for human consumption in a bid to promote spiritual purity [25]. For instance, on the day of atonement, no Jew is expected to eat anything for 24 h. Also, during the first nine months of “AV” they are not allowed to eat meat [26]. In line with spiritual purity, Islam prohibits consumption of pork and alcohol. Catholics are also not allowed to eat meat of Friday during the lent period. Food taboos in religions further aims at fostering and reinforcing religious identity. Adhering to certain food restrictions demonstrates devotion and commitment of individuals to their faith. Thus, religion plays an important role in influencing food choices. Cultural norms and customs govern dietary intake behaviours in several traditional societies comprising of critical life stages such as pregnancy [27]. Traditional cultural beliefs are known to create food taboos [28, 29] and barriers to vital maternal and child nutrition efforts in the first 1000 days [31, 32]. For example, in many cultures, mothers believe that respecting food-based taboos will result in a healthy pregnancy [33]. As reported in various studies, nutritious foods are usually avoided during pregnancy, owing to cultural taboos and misinformation [10, 32–34]. Cultural beliefs and taboos limit women’s ability to access some nutrient-rich food during pregnancy and breast-feeding. Many of the forbidden foods are abundant in essential trace elements, and their avoidance during critical stages of life could exacerbate trace element inadequacies [33].

Designing interventions to counteract the effect of hidden hunger among pregnant and breast-feeding women would require a clear understanding of the role of cultural beliefs and taboos on food availability and consumption in Buyende since they have been reported by Ara et al. [35] to be counterproductive in the adoption of micronutrient programs in low- and middle-income countries elsewhere. Prohibition of some foods during pregnancy stems from a belief that transgression of taboos may harm the mother or baby. Thus, any miscarriage or complications during birth or a baby being born with abnormalities are often believed to be caused by the mother who could have eaten certain foods not allowed in pregnancy. For instance, Gibbs [36] reported that sight loss and limb malformations were believed to be caused by broken taboos. However, food taboos and dietary restrictions contribute to high prevalence of low birth weight and hence the need to appreciate such taboos so as to construct educational policies that ensure provision of appropriate healthcare for pregnant women. This study examined the food taboos, cultural beliefs and nutritional misconceptions that influence diets of pregnant and breast-feeding women and thus the hidden hunger status in Buyende District. The study was based on the following research questions: (i) What are the cultural beliefs and food taboos related to nutrition in pregnancy? (ii) How do food taboos affect pregnant women food choices. It further hypothesized that low levels of education, poverty and limited access to nutritional information are major contributors to hidden hunger during pregnancy and breast-feeding. We envisage that the results from the study could inform the development of appropriate nutrition interventions that are cognizant of the cultural beliefs and taboos for wider acceptability by the affected communities. This could significantly contribute towards the attainment of sustainable development goal 2 (SDG-2) that among other things targets to end undernutrition most especially among the vulnerable groups.

## Methods

### Study area

**Fig. 1:**
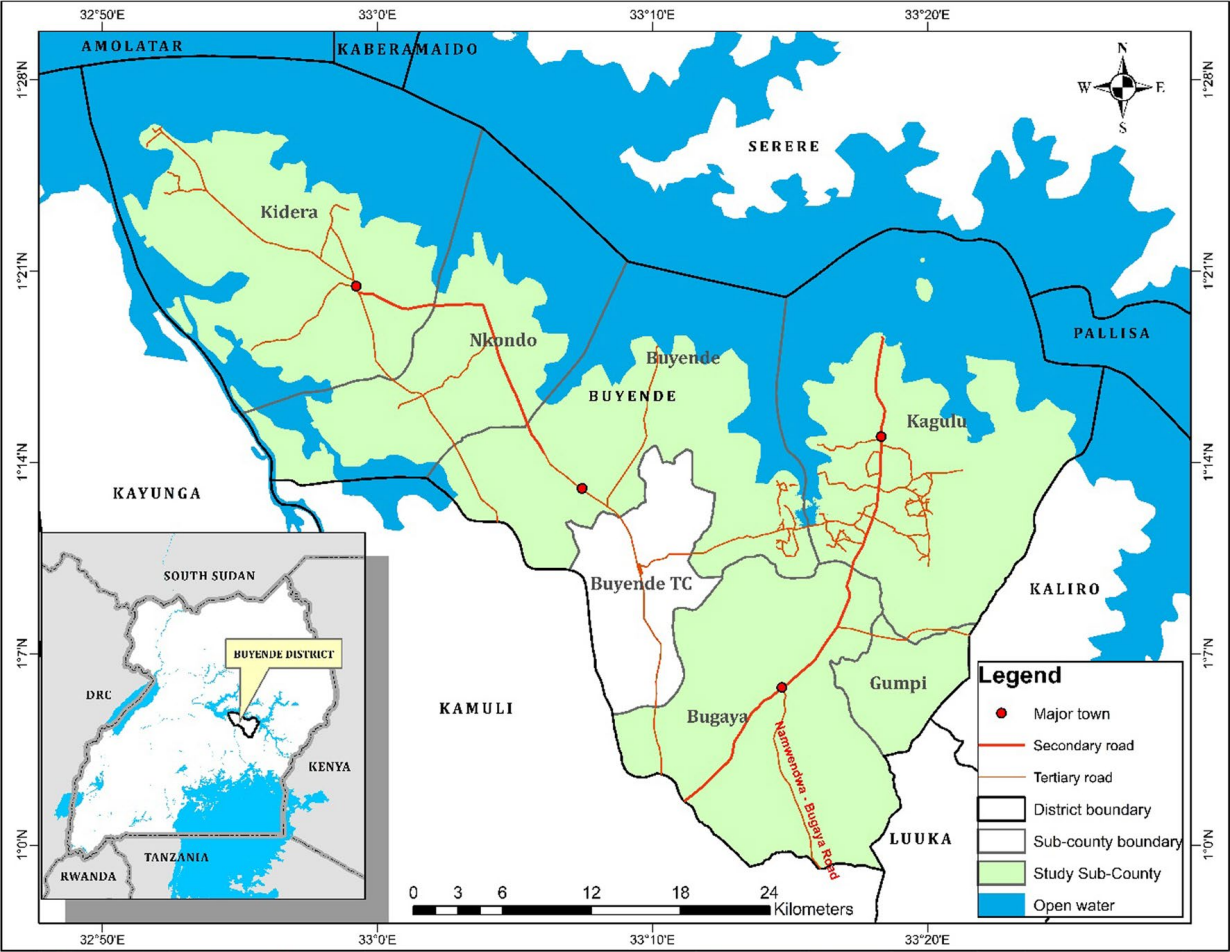
Map of the study site

Buyende district is located in the Busoga subregion in Eastern Uganda approximately 96 km from Jinja City and 180 km from the capital city Kampala [37]. The district, located in the cattle corridor, occupies a land area of 1,956 Km^2^ most of which is arable with an average elevation of about 1080 m above sea level [38]. It lies within latitudes 0045’N and 10 05’N and longitudes 33,047′E and 34,005′E (Fig. 1).

### Climate

Buyende experiences a tropical climate with an average annual rainfall of 160.7–237.1 mm that is bi-modally distributed with wetter periods occurring from March to May and August to November [39]. However, due to a high rate of deforestation and severe wetland degradation mainly due to the need for arable land, and charcoal burning as a source of income, the microclimate of the district has changed. It is now characterized by prolonged droughts, high temperatures and floods. The annual low temperatures range from 16.82 to 17.17 °C, while annual high temperatures range from 27.18 to 29.74 °C [39]. Agricultural production in the district is largely rain-fed, dominated by smallholder producers and characterized by low inputs, poor natural resources management and production systems which are highly vulnerable to climate change impacts [40]. Unreliable rainfall both in quantity and timing is still a big challenge to farmers. The adverse weather conditions have often resulted into crop failure and loss of livestock, hence contributing to increased households’ food insecurity and poverty [39]. It is estimated that over 30% of the people of Buyende suffer from chronic food insecurity annually [41, 42].

### Demographic characteristics of Buyende district

Buyende has a total population of 323,067 (61,199 households) with 49.1% (158,615) male and 50.9% (164,452) female [40]. About 42% of the population of Busoga where Buyende is located live below the poverty line, spending approximately $1.9/day and experiencing pockets of severe food insecurity [41]. It has a high teenage pregnancy rate of 25% that is higher than the national average (24%) and a fertility rate of 6.9 children per woman, which is also higher than the national average [43]. Other health burdens in the region include high maternal mortality, malaria incidence, parasite prevalence, malnutrition and anaemia, [41]. All these factors endanger lives of women in the reproductive age in the area which calls for intervention measures.

The local people are mainly of the Bantu ethnic group of the Basoga, Bagwere and Baganda tribes [26]. Up to 13% of females aged 12–17 and 22% aged 12–19 have given birth [42]. Crop growing is the main activity employing 94% of the households, with maize (85%) as the main crop [39]. The proportion of households engaged in growing of other crops is in the order: sweet potatoes (54%), beans (26%), millet (22%), matooke (8%) and coffee (2%). Almost three quarters (70%) of the households are engaged in livestock farming. Up to 86% of the households depend on subsistence farming as a main source of livelihood. Additionally, 5% of the households, aged 5 years and above, consume less than two meals in a day [44].

### Study design

A mixed methods approach that included both quantitative and qualitative techniques. Field study was conducted between May and July 2023. A cross sectional survey with purposive sampling method was employed in enrolling study participants to obtain quantitative data. Pregnant and breast-feeding women presenting to the health centres and who consented to participate in the study were enrolled. They were interviewed on their beliefs and food taboos during pregnancy and while breast-feeding. The interview was done using a structured pretested questionnaire. All interviews were conducted in Lusoga, the respondents’ local language, and in some cases, in English. Both translators and enumerators were trained on how to conduct interviews using the questionnaire, so as to provide full understanding of the questions administered.

In order to obtain qualitative data, focus group discussions (FGDs) and key informant interviews were conducted. Focus group discussions and key informant interviews complemented data from questionnaires. Each focus group comprised of 6–10 members and a total of 8 FGDs were conducted. A total of 15 key informant interviews were conducted. These added more insights into food taboos and cultural beliefs among pregnant and breast-feeding women in Buyende district. These enhanced the understanding of the perceptions regarding food taboos and cultural beliefs that affect pregnant and breast-feeding women.

### Quantitative surveys

**Fig. 2:**
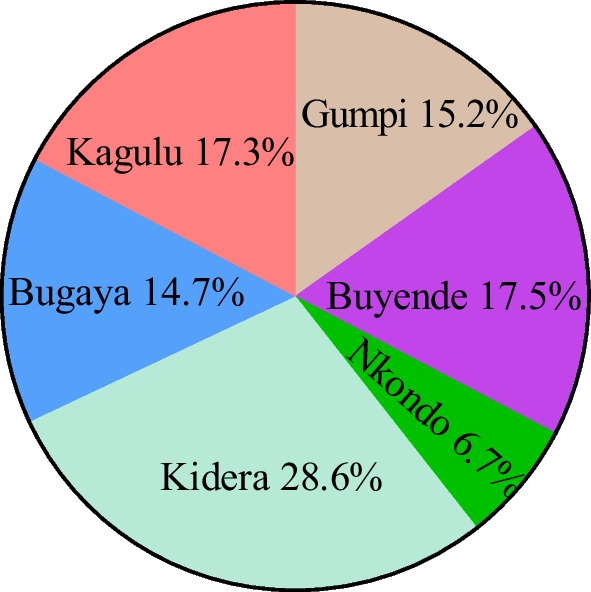
Percentage of respondents per sub-county

All the sub-counties of Buyende district (Bugaya, Buyende, Gumpi, Kagulu, Kidera and Nkondo) formed part of the study (Fig. 2). Before fieldwork, a consultative meeting with district officials who included: the Resident District Commissioner, the Chief Administrative Officer, District Internal Security officer, District Health Officer and in-charges of all health centres in Buyende district was conducted. The purpose of the meeting was to seek permission to conduct the research from the district administration, build consensus on various aspects of the research, seek for security during the research activity and obtain first-hand information on the hidden hunger situation in the district. The study was conducted at 29 health centres (II, III and IV) within the sub-counties targeting pregnant and breast-feeding women that visited for different health services such as antenatal care and immunization and all who consented formed part of the study participants.

All women that visited the health facility and consented to participate in the study on the given days were interviewed in order to obtain information about the food taboos and cultural beliefs they experience. At the end of the study period, 462 women had been interviewed in the six sub-counties as indicated by proportions in Fig. 2.

The questionnaire included some open-ended questions on food taboos and cultural beliefs among pregnant and breast-feeding women, especially related to avoidance or consumption of some foods due to cultural beliefs (food taboos) and reasons advanced for such. Participants were also asked to provide information from where such knowledge was obtained. Women were asked to list the various food items that were associated with taboos and cultural beliefs in their communities and reasons for avoiding the foods during pregnancy or breast-feeding. Voucher specimens for all mentioned plants were collected, processed and taken to Makerere University Herbarium for identification. Correctness of scientific names was checked in the database of Flora of the World https://powo.science.kew.org/accessed on 15 September 2023.

### Key informant interviews

Key informant interviews were conducted at some health centres with the following personnel; nutrition focal person, midwife in-charge and health centre administrator. The key informants provided insight into best practices for nutrition. In total, 15 interviews were conducted.

### Focus group discussions

Focus group discussions (FGDs) were conducted to attain group interaction on the issues relevant to the study [45, 46]. Seven FGDs were conducted with women of mixed age groups, while one FGD was conducted with elderly men each with 6–10 participants. Data saturation (determined the number of FGDs) was reached when no new additional information was obtained. There were 61 participants in total, and these were purposively recruited by the community leaders and officers in charge of health facilities, who also helped with organizing the venues and times for the FGDs. Participant selection targeted those who had not participated in the health centre surveys. The composition of the study participants was diverse with regard to age, pregnancy status and whether the participant was a mother. The FGD with men participants was conducted in Kimbaya Village, Bugaya sub-county, to triangulate the information provided by women. Sex-specific groupings (females only) were done to ensure that males did not hinder the freedom of female participants to express opinions and openly discuss what are largely deemed as women’s issues. Participants were given numbers, helping the note-taker to recognize who was responding, anonymously. The FGDs were held in a quiet, comfortable and convenient setting and each FGD took between 1 and 1½ h. To capture all the information, voice recorders were used and also field note-taking was done. This was done by both the member of the research team and a research assistant (health worker), after consent was provided by the participants. We obtained information on the food taboos and cultural beliefs among pregnant and breast-feeding women in Buyende district and reasons advanced for these. At the end of each day, the field notes were expanded by the note-taker with assistance from the moderator while creating a daily report on data obtained. Thereafter, comprehensive verbatim transcription of the records was performed.

In order to minimize language barrier challenges, research assistants knowledgeable in Lusoga language, culture and its norms were used in the study. All questions asked in the FGDs were open-ended, with new questions arising from the responses given, as participants were able to build on each other’s ideas and comments. During FGDs, prompting questions about the main themes of taboos and cultural beliefs that influence food choices for pregnant and breast-feeding women, prohibited and preferred foods and reasons for the practices were asked.

## Data analysis

### Quantitative data analysis

Data were entered and cleaned using Microsoft Excel, and descriptive statistics were obtained using Statistical package for social Scientists (SPSS) version 25. Descriptive data were presented as means and standard deviations (SDs) (mean ± SD) and percentages.

### Qualitative data analysis

At the end of each FGD session, the researcher and the assistant compiled their notes into one document, thus linking accurately the statements to anonymously-coded individual identifiers in each group. Data from all the FGDs in field notes and on the voice-recorder were translated from Lusoga to English. Qualitative data were manually coded and categorized into main emerging themes [47]. Themes were then analysed through coding [45]. Most sections of the discussions were quoted verbatim to increase readability. The verbatim transcripts were reviewed for completeness and quality control.

## Results

### Quantitative assessments

#### Socio-demographic characteristics of pregnant and breast-feeding women

This study had a total of 462 respondents who were either pregnant (256, 55.4%) or breast-feeding (206, 44.6%). Of all the 462 women interviewed, 129 (27.9%) agreed to practicing food taboos and cultural beliefs. The mean household size for all the respondents was 5.3 people. The respondents were from all the 6 sub-counties of Buyende district (Fig. 1) with majority from Kidera sub-county (28.6%), followed by Buyende sub-county (17.5%) and Kagulu sub-county (17.3%). Among the age categories, individuals aged 21–30 years constituted the largest group, accounting for 48.1% (222) of the respondents, while 162 (35.1%) of the respondents were aged between 31 and 40 years (Table 1). Furthermore, the study revealed that majority of the respondents (346, 74.9%) had only completed primary education, while 77 (16.7%) of them had accomplished secondary education. Only 15 (3.2%) of the respondents had tertiary education qualification (Table 1). The study population predominantly comprised of married individuals, with 88.1% representation.

**Table 1:** Socio-demographic characteristics of respondents (N = 462)

Socio-demographic factors	Percentage (N = 462)	Socio-demographic factors	Percentage (N = 462)
Age category		Marital status	
< 20	6.3	Divorced	1.1
21–30	48.1	Married	88.1
31–40	35.1	Single	0.9
41–50	10.6	Widowed	9.3
		Separated	0.6
Education level		Religion	
No formal	5.2	African tradition	0.2
Primary	74.9	Anglican	26.6
Secondary	16.7	Catholic	30.1
Tertiary	3.2	Muslim	18.8
Occupation		Orthodox	0.9
Civil servant	1.1	Pentecostal	14.1
Farming	85.5	Seventh-day Adventist	4.3
Trading	3.5		
Fishing	0.2		
Others	9.7		

### Cultural beliefs and food taboos during pregnancy

The most commonly-known tabooed foods among pregnant women within Buyende district included sugarcane (17.8%), fishes (15.2%), oranges (6.6%), pineapple (5.9%), eggs (3.3%) and chicken (3.3%) (Table 2). Other foods were cited as taboo foods during pregnancy but were only mentioned by less than 3% of women in each case. Several reasons were advanced for not consuming taboo foods which ranged from issues that may affect the mother during delivery, breast-feeding to health issues mostly affecting the child. Others included cultural issues that could hardly be explained since prohibition of a certain food was generally accepted by the culture. These reasons are detailed in Table 2, together with information on where the knowledge on food taboos was acquired. Knowledge on the taboo foods and the consequences of consuming these foods was primarily acquired from all members of the community such as family members, especially grandmothers and mothers, health workers, elders, fellow women and village health teams. However, some women reported that they observed a food taboo because of their own experience from previous pregnancies.

**Table 2:** Food avoidance due to taboos and cultural beliefs among pregnant women in Buyende district

Scientific name (Voucher No.)	Local name (language)	Respondents (%)	Reasons for prohibition	Medical term for prohibition	Source of information
Plant based food					
*Amaranthus dubius* Mart (PT010)	Doodo (Lug)	0.7	Causes early contraction	Preterm labour	Mother
*Ananas comosus* (L.) Merr (PT001)	Enanhasi (Lus)	5.9	Increases saliva production Causes abortion and wounds in baby’s head	Drooling	Elders, mother in law
*Arachis hypogaea* L. (PT20)	Amaidho (Lus)	0.7	Causes skin disorder		Friend
*Artocarpus heterophyllus* Lam (PT002)	Feene (Lus/Lug)	1.3	Causes overweight baby and difficulty during child birth Increase in saliva production	Foetal macrosomia, Drooling	Grandmother
*Capsicum frutescens* L. (PT011)	Kalaali (Lus)	2	Reduces breastmilk production Causes congenital eye problems	Hypogalactia	Community Mother-in-law
*Citrus limon* (L.) Osbeck (PT003)	Enniimu (Lus)	0.7	Causes underweight baby	Intrauterine growth restriction	Elders
*Citrus sinensis* (L.) Osb. (PT005)	Mucungwa (Lus)	6.6	Causes skin diseases, Excessive secretion of saliva Shortness of breath Baby will not grow hair	Drooling Dyspnoea Short anagen hair syndrome	Community. Mother
*Cleome gynandra* L. (PT004)	Yobyu (Lus)	2.6	Causes abortion		Grandmother Aunt
*Corchorus olitorius* L (PT009)	Mutele (Lus)	1.3	Increased saliva production	Drooling	Community
*Dioscorea bulbifera* L. (PT008)	Amasoma(Lus)	3.0	Causes the mother’s skin to itch Reduces breast milk May cause death of baby	Hives Hypogalactia Neonatal death	Herself Community Grandmother
*Eleusine coracana* Gaertn (PT006)	Millet & millet porridge (Eng)	2.7	Diarrhoea, Makes the baby to have a very big head	Foetal macrosomia	Community, mother
*Glycine Max (L) Merr.* (PT007)	Soy beans (Eng)	0.7	It will cause the umbilical cord to wrap the baby’s neck	Nuchal cord	Friends
*Hibiscus sabdariffa* L. (PT012)	Kimuli ekimwifu (Lus)	0.7	It is not good for the baby		Mother
*Ipomoea batatas* (L.) Lam**. **(PT013)		2.0	Causes diarrhoea, reduces energy		Community, Elders
Maize porridge		0.7	Baby is born with rough skin		Community
Mixture of maize and beans	Empengeere (Lus)	0.7	Development of an enlarged umbilical cord		Mother in-law
*Momordica foetida* Schumach (PT014)	Mabombo (Lus)	1.3	Causes abortion	Abortifacient	Community
*Musa acuminata* Colla (PT019)	Bogoya (Lug)	0.7	Causes death when eaten		Grandmother
*Oryza sativa* L (PT016)	Rice (Eng)	1.3	It causes itchy skin and heart burn to the pregnant woman A big baby causing difficult delivery	Foetal macrosomia	Community
*Phytolacca dodecandra* L’ Her (PT017)	Nyante (Lus)	0.7	It causes abortion	Abortifacient	Elders
*Saccharum officinarum* L. (PT018)	Ekikajo (Lus/Lug)	17.8	Production of a lot of saliva in baby Leads to water in the womb & causes cough Causes a big baby that becomes hard to push during delivery	Drooling Foetal macrosomia	Mother, fellow women, elder, grandmother, community
*Solanum anguivi* Lam. (PT021)	Endagi (Lus)	0.7	Causes skin rash to the baby		Elders
*Solanum lycopersicum* L. (PT022)	Enhaanha (Lus)	0.7	Spoils baby’s skin, causes general weakness		Community, mother
*Solanum nigrum* L (PT023)	Eilibwa (Lus)	0.7	Causes curse		Elders
*Solanum gilo* Raddi (PT024)	Scarlet eggplant (Eng)	2.0	Causes the baby’s skin to itch	Hives	Community, Aunt, mother-in-law
*Sesamum indicum* L. (PT029)	Mukose (Lus)	0.7	Increases saliva production	Drooling	Hear say
*Tamarindus indica* L. (PT025)	Nkooge (Lus/Lug)	0.7	Causes sores on the body of mother		Mother
Tea leaves		0.7	Baby becomes too big to push during delivery	Foetal macrosomia	Community
*Vigna subterranea* (L.) Verdc)(PT026)	Kulekula/mpande (Lus)	0.7	Causes the baby’s head to become too big	Macrocephaly	Mother
*Vigna unguiculata* L. (PT027)	Egobe (Lus)	0.7	May cause death of baby	Neonatal death	Grandmother
*Zingiber officinale* (Roscoe) (PT028)	Ginger	0.7	Induces labour	Oxytocic	Community
Animal food items					
Chicken		3.3	Culturally women are not supposed to eat		Elders
Eggs		3.3	Baby too big causing difficulty in delivery Uncoordinated movement of the baby	Foetal macrosomia Apraxia	Elders
Liver		1.3	Speech disorder to the baby		Elders
* Rastrineobola argentea*	Mukene (Lus/Lug)	12.0	Taboo		Ancestors
Animal food items					
Pork		1.3	Religious reasons for Muslim women		Parents,
Rabbit meat		1.3	Reduces breast milk production	Hypogalactia	Community, elders
Yoghurt		0.7	Causes overweight baby	Foetal macrosomia	Mother
Other items					
Salt		2.0	Spoils baby’s skin and eyes		Mother-in-law
Sugar		1.3	Increases saliva production (Ptyalism) Causes cough		Community, mother
Sour foods		0.7	Affects the eyes		Elders
Water		0.7	When you take water after delivery you die Too much water after delivery hampers the healing of the stitches and slows internal healing too		Community

Language: Lus = Lusoga; Lug = Luganda; Eng = English. Other items in the column of food name include other items consumed which cannot be classified as animal or plant based. Medical terms for prohibition reasons were interpretation of authors not given by respondents

### Qualitative assessments: focus group discussions and key informant interviews

Major themes that emerged from FGDs regarding food taboos and cultural beliefs were: tribal prohibitions, religious prohibitions and prohibitions by spirits. Some participants in the FGDs admitted to existence of food taboos and cultural beliefs during pregnancy and breast-feeding, while others were not aware of any taboos reasoning that they existed long ago and are no longer applicable. A case in point was elderly women from Mpondwe health centre who revealed that they no longer have tribes that restrict women from eating a particular type of food or sauce except Moslems that do not eat pork. One elderly woman from this FGD emphasized that

back in the days, they used to restrict women from eating mudfish, but these days we eat”. Another one retorted that “in our years they used not to allow us to eat chicken but these days, women love chicken like nothing.

Participants in this FGD reported that there were no specific foods that pregnant and breast-feeding women were prohibited from eating. They further revealed that it was only pure original Balalo (traditional cattle keepers) that never ate chicken but they are no longer inhabiting the area. One participant said that

it is only when her pregnancy does not like that particular food but otherwise, they are not restricted.

Another responded that

also may be when she cannot access that food that’s when she may not eat it.

Apart from the FGD of elderly women in Mpondwe, all other FGDs revealed foods that were mentioned in the survey and gave the same reasons for being prohibited during pregnancy or breast-feeding. The foods mentioned include: chicken, millet, sugarcane, mushrooms, duck, white ants, hard corn, pineapple in addition to excessive sugar and salt. There was a strong emphasis on fish as a taboo food especially among the Seventh-day Adventist (SDA) and Bachwezi tribe from all FGDs. For instance, a breast-feeding woman from Kidera said that,

on the side of lactating mothers who are SDA, they are told not to eat catfish and yet, if its soup is taken can lead to production of more breast milk.

Participants from all FGDs also stated that the Bachwezi do not eat fish. An elderly man from Kimbaya village said that,

I can’t talk so much about culture but religion because this side, we have religions that prohibit a person from eating fish or anything from water yet these things have nutrients that can build a woman’s life and the baby as well. A woman misses such food values. Regarding culture, our side here, there are people who call themselves the Bachwezi, they stop people from eating silver fish yet they would have eaten and helped strengthening of baby bones and those of the pregnant woman as well.

Another food taboo that arose from FGDs was that consumption of sugarcane or excessive sugar that were believed to cause drooling to the baby. The same information was revealed by the survey. An adolescent mother from Irundu health centre said that,

I have heard that eating sugarcane when you are pregnant can make a baby to drool.

Another adolescent mother from Irundu said that

they say also pineapples when you eat pineapples when you are expecting, you will produce a drooling baby.

Regarding millet, a midwife from Bukungu health centre during an interview revealed that,

like when a mother is pregnant, she does not eat millet or take millet floor. They say that when they take and they deliver, that baby dies.

Prohibition of millet and millet flour were reported in the Bukenye tribe and one participant reported that.

But in the Bakenye tribe, a newly delivered mother cannot eat millet Bwita before the baby’s cord drops off.

All participants in the group agreed that this belief is still followed up to today.

All participants from FGDs who mentioned chicken as a taboo food revealed that its consumption causes rash. This was reported in mainly two tribes of Bachwezi and Banyankole. For instance, a teenage mother from Buyende health centre III said that.

when I ate chicken, I developed rash-like things that are on chicken but there were herbs I was bathed with and they ceased, I now eat.

Another teenage mother also said,

for us as a tribe (Bachwezi), we don’t eat chicken but when I got pregnant, they said, I eat it and it has no issue. Right now I eat.

In the same group another participant said

I can be here when I am a Muchwezi and they restricted me from eating chicken, now when I eat it the other things(spirits) will come and hit me, I then start developing rashes because of eating chicken.

Regarding mushrooms, a teenage mother said that

there are tribes that do not eat mushrooms (small type), so when you reach a man’s clan who doesn’t eat those mushrooms, automatically you don’t eat them. If you eat them definitely the foetus has to come out.

When probed further whether such an incidence has ever happened, she said that,

yes, it occurred. There is a lady, I can say she is my inlaw, for us we don’t eat red mushroom but for her she went and looked for that mushroom and ate it, the baby came out.

## Discussion

With the low levels of education, poverty and limited access to nutritional information, we anticipated a higher adherence to food taboos among pregnant and breast-feeding women in Buyende. The study revealed a 27.9% prevalence of food taboo practices among pregnant and breast-feeding women. This prevalence was similar to that of 27.5% reported in a recent study by Abere and Azene [48] among pregnant women attending antenatal clinics, in North West Ethiopia and lower than the 67.4% reported by Mengie et al. [49] among agro-pastoralist pregnant women in Ethiopia. The percentage could be higher than what is revealed by this study since the average age of the respondents was below 35 years, above which according to Zepro [16], and Getnet et al. [50] women are more likely to practice food taboos. Furthermore, the lower prevalence could be ascribed to that fact that respondents were mainly women seeking for antenatal care from health centres. Food taboo practices among such women has been reported to be low by Mengie et al. [49].

There is observance of food taboos among pregnant and breast-feeding women in Buyende district. These taboos are anchored on tribal, religious beliefs and selfish patriarchal tendencies in some African cultures [18]. Like in western Bengal knowledge about food taboos is upheld and enforced by mothers-in-law, aunts and other elderly female relatives [51]. Adherence to food taboos and cultural beliefs collaborates results of other studies conducted in countries like Indonesia [32], Thailand [17], India [52] and China [53] in Asia as well as in African countries like Ghana [54], Ethiopia [55], Nigeria [56], Sudan [57] and Uganda [58]. The main reason for not consuming the taboo foods was attributed to the health consequence on mothers themselves, such as abortion and difficulty during labour and also to safeguard the birth outcomes of the babies, similar to what was believed in other cultures [59–61].Thus, the prohibited foods are those considered problematic for the mother, foetus or baby. A normal mother instinctively and unconditionally evolves love for her child and would do anything in her power to protect her child from maltreatment. So if a general belief in society is that consumption of a specific food would result in a child with abnormalities, mothers would instinctively avoid such a food. Unfortunately, many cultural beliefs and food taboos involve foods that are known to be rich sources of protein, iron and micronutrients, all of which are critical during pregnancy [62], and often not consumed in sufficient quantities by women. Although the women who practice these food taboos may have less nutrient intake, it does not necessarily mean they are hungry. They may eat larger quantities of other less-nutritious food rich in calories but deficient in minerals and vitamins which can fill their stomach barring the hunger feeling [63]. Thus, hidden hunger symptoms are not obvious and people may not be aware of it.

Foods were tabooed to avoid labour complications and undesirable health effects on the baby. Some animal-product foods especially fish and eggs in addition to beans, which are rich in proteins, vitamins and mineral nutrients were avoided for the fear of the mother discharging a foul smell from the vagina, baby becoming too big resulting in a difficult delivery and causing diarrhoea.

This in conformity with earlier findings reported by [53] in the study that was conducted in Ghana. Eggs were avoided owing to the conviction that they cause overweight in the foetus, which contributes to difficulties during child birth. A big baby often may lead to prolonged labour exposing both mother and baby to potential complications. Prolonged labour is associated with an increased risk of having a caesarean birth which is expensive and not affordable by poor women. Most often health centres in rural areas do not have operational theatres implying referrals in case emergencies arise. These risks contribute to adherence to some taboos to avoid very big babies.

However, scientific evidence revealed that eggs contain fatty acids, proteins, choline, vitamins A and B12, selenium and iodine in amounts higher than that found in other animal food source [64]. Some of these nutrients in eggs are critically needed to support the health of the pregnant and breast-feeding mothers and foetus. For example, iodine is essential for the production of thyroid hormones, thyroxine (T4) and triiodothyronine (T3), required for the control of metabolic processes and growth and development, especially of the brain and central nervous system up to 3 years of age [65]. During pregnancy and breast-feeding iodine has also been reported to aid neuronal proliferation, growth of axons and synapses, as well as myelination of neurites [66–68]. Thus, such taboos that precludes the consumption of eggs may contribute to the persistence iodine dietary deficiency or hidden hunger that has been reported by [69] to be rampant in poverty-stricken communities, such as Buyende. An empirical study revealed that inadequate intake of chlorine during pregnancy was associated with neural tube defects and misfunctioning of the child’s brain [70]. Thus, consumption of eggs during pregnancy has potential of improving childbirth outcomes and promoting brain development. Vitamin D in eggs is important in protecting the newborn baby from development of rickets and promotes healthy teeth and bones of the mother [68]. Despite the importance of eggs in the diet of pregnant and breast-feeding women, consuming them excessively is associated with high cholesterol content which can potentially lead to cardiovascular diseases [71]. Additionally, egg consumption has been associated with adverse health effects due to possession of antinutritional factors like ovomucoid that can inhibit trypsin that bind biotin [72]. Also, egg and egg-derived foods can result in food borne illnesses mainly caused by *Salmonella* [72]. Another health-related risk to egg consumption is the potential presence of residues of veterinary drugs used in treatment of laying hens in the eggs [73]. Thus, whereas eggs possess nutrients that are beneficial to pregnant and breast-feeding women, their avoidance through adhering to taboos and cultural beliefs protects women from adverse effects of their excessive consumption.

Most foods in the current study were also tabooed to protect a child from being born susceptible to many diseases, including skin rash and eye diseases plus other conditions like excessive production of saliva and being under weight. Pineapples, oranges and sugarcane are not consumed to avoid excessive secretion of saliva, difficulty in breathing and loss of hair in babies. However, restricted consumption of such fruits which are rich in vitamins A and C denies the pregnant women these vital nutrients. A similar study in Uganda revealed that pregnant women were prohibited from consuming milk, eggs, meat, sugarcane and organ meat [57]. Studies in other African countries also reported the avoidance of some of these foods during pregnancy though the reasons that were given differ from those in the current study. For example [16] reported the avoidance of oranges and pineapples in Ethiopia for reasons linked to having a baby with discoloured skin; inducement of abortion or stillbirth; and to protect the baby from contracting worms, malaria and diarrhoea during childhood. In South Africa, pineapples were avoided because they would cause the baby to have a cracked skin that would be difficult to treat [74] closely related to a reason of causing wounds in a baby’s head in the current study. On the contrary consumption of pineapples during pregnancy were recommended in India with a belief that it leads to production of a clean baby [75]. Cherkose et al. [76] reported the avoidance of potatoes by pregnant women to avoid having big babies, which can cause labour difficulties. Excessive production of saliva by a baby can cause discomfort and embarrassment to the mother since traditionally such a baby will be regarded to be mentally retarded.

Fish which was majorly restricted in consumption by pregnant women in the current study was also a taboo food during pregnancy in Tanzania [74], Indonesia [32] and Malaysia [59], as it was believed to cause difficulties during childbirth. Whereas some foods such as meat, fish, chicken, eggs, and milk were recommended as being beneficial during pregnancy in Egypt [77], they were restricted in the current study. In Buyende, eggs were avoided due to a belief that they make the unborn fat resulting in difficulty by the mother to push during delivery or that the child will not talk, while a study in Tanzania and other parts of Africa revealed the reason for eggs avoidance as fears related to the animal’s characteristics being transferred to the child or sterility [78]. In fact, there is empirical evidence in support of intake of animal proteins to improve birth outcomes especially to increase neonatal birth weight [79]. Thus, pregnant women who tabooed eggs might have been at risk of lacking enough sources of proteins that could result in underweight babies who often exhibit a lot of complications.

Sugarcane and pineapple featured prominently among the tabooed foods in Buyende where it was believed that their consumption results in excessive production of saliva by a baby, increased water in the womb, cough, an overweight baby that becomes hard to push during delivery, abortion and wounds in a baby’s head. Similarly, a study by Mohamad and Ling [80] by in Kuala Lumpur reported that pineapple and sugar cane were regarded as taboo foods by more than half of subjects. However, reasons for avoidance in this case were fear of abortion, excessive bleeding during labour, deformed babies, difficult labour, vomiting and oedema. The abortive effect of pineapples was also reported in Malaysia by [81] where it was regarded as a hot food capable of causing strong uterine contractions. Debnath et al. [83] reported that excessive consumption of fresh pineapple juice can cause mouth and oesophagus soreness and if consumed on an empty stomach can result in stomach upset. Additionally eating too much pineapple may contribute to gingivitis and cavities. Also too much consumption of pineapple fruit during the second trimester of pregnancy was associated with an increased likelihood of gestational diabetes mellitus [83]. Therefore, avoidance of pineapples during pregnancy may accord health benefits to the mother.

Avoidance of rice during pregnancy was reported in similar studies in Ethiopia, Nigeria, the Central African Republic and Tajikistan in India [12, 16, 28, 84] with the aim of suppressing gestational weight gain (GWG) which is associated with labour complications as a result of baby macrosomia. This could be because most of the reasons advanced for avoiding food have no scientific validation. This therefore calls for nutritional education regarding the food values important during pregnancy and their food sources to avoid being misled by taboos. However, it should be noted that though there is high maternal and neonatal morbidity and mortality in Uganda [85], their occurrence cannot be merely be attributed to food taboos and cultural beliefs. Maternal mortality is attributed mainly to haemorrhage, obstructed labour, pregnancy-induced hypertension, unsafe abortions, and septicaemia [85]. Another primary obstacle to healthcare in Uganda is accessibility, as individuals live a mean of 3–5 km from the nearest health facility [86] which hinders women from seeking antenatal and postnatal services and thus likely to contribute to morbidity and mortality of both mothers and their children. Other major bottlenecks in the provision and use of maternal and childcare services were reported as delays in deciding to seek care, reaching points of care and receiving quality care at points of delivery [87].

Apart from food taboos, there are also several wild foods recommended to pregnant women in sub-Saharan Africa [88]. For instance, some wild vegetables are rich in different nutrients which are important in the nutrition of pregnant and breast-feeding women but often overlooked and regarded as poverty foods, yet they would be easily accessible and affordable to the poor rural women. *Nephrolepis biserrata* is rich in iron, while soup from a mixture *of Ricinodendron heudelotii* and *Elaeis guineensis* is rich in proteins. Women in Ghana and Benin cited provision of strength as the main reason for consumption of wild plants [89]. Wild plants are often neglected and underutilized yet they contribute to food and nutrition security since they are highly adapted to climate change and contain high micronutrient content. Therefore, wild foods are instrumental in curbing hidden hunger. However, caution should be taken while consuming a vegetarian diet only as such is deficient of Vitamin B12 and Zinc whose main source is animal products [90]. Fikkari et al. [91] study revealed low body weight in infants born to mothers who adhered to vegetarian diet. High intake of fruits and vegetables with low or no intake of meat and fish which are sources of nitrate, nitrite and N-nitroso compounds is associated with a higher risk of neural tube defects [92]. Iron deficiency during pregnancy has been associated with low birth weight and neonatal anaemia [93]. This is because iron derived from meat has better bioavailability than iron obtained from plants. Thus, women that are vegetarians ought to take a higher content of iron supplements to avoid depletion of internal sources [94]. In view of the above discussion, appropriate and effective health education on food during and after pregnancy should focus on the underlying reasons for food taboos and the recommended (wild) foods during pregnancy. Thus, women who do not want to eat fish during pregnancy because of their beliefs could substitute it by consumption of more of the preferred protein-rich vegetable that is recommended during pregnancy within the same cultural belief system and supplement this with iron.

## Conclusions

The present study revealed that nearly 27.9% of the respondents practice food taboos and adhere to cultural beliefs related to their dietary habits during pregnancy and breast-feeding that are fuelling the prevalence of hidden hunger. This finding underscores the significance of addressing these cultural factors in maternal and child nutrition interventions. The mean age of the women participating in the study was 31 years, suggesting that these practices are adopted by very young pregnant and breast-feeding women, which further exacerbates the potential impact of these food taboos to maternal and child health. Furthermore, the study area was characterized by a high mean household size (5.3 persons), high teenage pregnancy rate (25%), poor economic status and low level of education, which further highlights the potential impact on maternal and child health. Additionally, our study revealed that the most tabooed foods among pregnant women were sugarcane, fishes, oranges, pineapple and eggs. However, most of the foods prohibited during pregnancy and breast-feeding have been reported in empirical studies to contain a number of food values necessary to promote the health of both the mother and guard against deformities in the unborn child, and also to provide strength for a breast-feeding mother and to transfer the required nutrients to the baby. Therefore, these dietary restrictions have implications for the nutritional status and overall health of pregnant women, bread feeding women, the foetus and children. Major reasons advanced for avoidance of such foods included excessive saliva production by baby, a big baby that will cause difficulties during delivery, vaginitis after consumption of fish, skin diseases, breathing difficulties to the baby and abortion. This diversity in reasons for these food taboos underscores the complexity and multifaceted nature of cultural beliefs and practices surrounding dietary choices during the maternal and child-rearing period. Notable some of the reasons mentioned for not consuming taboo foods were linked to deeply ingrained cultural beliefs that were difficult to explain. Therefore, there is need for a sensitive and culturally informed approach when addressing these practices. This calls for health education programs regarding nutrition which will in turn improve the maternal and child health. We suggest continued maternal education to the general public on these matters by designated health personnel in the district. There is also need to validate the claims so as to address the nutritional deficiencies and demystify the myths which could be caused by such beliefs.

## Data Availability

Data generated or analysed during this study are included in this article and are available from the corresponding author on reasonable request.

## Abbreviations

BHF: Busoga Health Profile
FGD: Focus group discussion
GWG: Gestational weight gain
ICF: Inner city fund
REC: Research Ethics Committee
SD: Standard deviation
SDA: Seventh-day Adventist
SDG: Sustainable development goals
SPSS: Statistical Package for Social Scientists
UBOS: Uganda Bureau of Statistics
UCU: Uganda Christian University
USAID: United States Agency for International Development
WHO: World Health Organization
